# Development and Psychometric Validation of Tinnitus Qualities and Impact Questionnaire

**DOI:** 10.3390/clinpract15050087

**Published:** 2025-04-27

**Authors:** Vinaya Manchaiah, Gerhard Andersson, Eldré W. Beukes, Marc A. Fagelson, De Wet Swanepoel, Eithne Heffernan, David Maidment

**Affiliations:** 1 Department of Otolaryngology-Head and Neck Surgery, University of Colorado School of Medicine, Aurora, CO 80045, USA; dewet.swanepoel@up.ac.za; 2 UC Health Hearing and Balance, University of Colorado Hospital, Aurora, CO 80045, USA; 3 Virtual Hearing Lab, Collaborative Initiative Between University of Colorado School of Medicine and University of Pretoria, Aurora, CO 80045, USA; eldre.beukes@aru.ac.uk; 4 Department of Speech-Language Pathology and Audiology, University of Pretoria, Pretoria 0001, South Africa; 5 Department of Speech and Hearing, Manipal College of Health Professions, Manipal University, Manipal 576104, Karnataka, India; 6 Department of Behavioral Sciences and Learning, Department of Biomedical and Clinical Sciences, Linköping University, 581-83 Linköping, Sweden; gerhard.andersson@liu.se; 7 Department of Clinical Neuroscience, Division of Psychiatry, Karolinska Institute, 717-77 Stockholm, Sweden; 8 Vision and Hearing Sciences Research Group, School of Psychology and Sports Sciences, Anglia Ruskin University, Cambridge CB1 1PT, UK; 9 Department of Audiology and Speech-Language Pathology, East Tennessee State University, Johnson City, TN 37614, USA; fagelson@mail.etsu.edu; 10 Audiologic Rehabilitation Laboratory, Auditory Vestibular Research Enhancement Award Program, Veterans Affairs Medical Center, Mountain Home, TN 37684, USA; 11 National Institute for Health and Care Research (NIHR), Nottingham Biomedical Research Centre, Nottingham NG7 2UH, UK; eithne.heffernan1@nottingham.ac.uk; 12 Hearing Sciences, Mental Health and Clinical Neurosciences, School of Medicine, University of Nottingham, Nottingham NG7 2QL, UK; 13 School of Sport, Exercise and Health Sciences, Loughborough University, Loughborough LE11 3UT, UK; d.w.maidment@lboro.ac.uk

**Keywords:** tinnitus, tinnitus sensation, outcome measures, psychometric validation, questionnaire

## Abstract

**Background:** To develop and validate the Tinnitus Qualities and Impact Questionnaire (TQIQ), a new tool for evaluating the perceived qualities of tinnitus sound. **Method:** The study was part of two clinical trials on internet-based tinnitus interventions, using cross-sectional (*n* = 380) and pretest–posttest data (*n* = 280). Participants completed various questionnaires online, including the newly developed TQIQ and measures of tinnitus severity (Tinnitus Functional Index; TFI), anxiety (Generalized Anxiety Disorder 7; GAD-7), depression (Patient Health Questionnaire 9; PHQ-9), insomnia (Insomnia Severity Index; ISI), and health-related quality of life (EQ-5D-5L Visual Analog Scale; VAS). The psychometric properties of the TQIQ were assessed, including construct validity, internal consistency reliability, floor and ceiling effects, interpretability, and responsiveness to treatment. **Results:** Exploratory factor analysis resulted in two factors that accounted for 57% of the variance—internal and external tinnitus qualities. Overall, 92% convergent validity predictions were confirmed; TQIQ total scores strongly (≥0.6) or moderately (0.30 to 0.59) correlated with the TFI, GAD-7, PHQ-9, and ISI. The known-groups validity prediction was confirmed as individuals with an overall TFI score > 50 (severe) obtained significantly higher TQIQ scores. All internal consistency reliability statistics were within the required range (Cronbach’s α > 0.8). Floor and ceiling effects were negligible. ROC established clinically important cut-off scores, enhancing the interpretability of tinnitus severity classification. Finally, 89% convergent validity predictions were confirmed; TQIQ and TFI change scores were moderately correlated, indicating good responsiveness of the former to treatment. **Conclusions:** The TQIQ has adequate psychometric properties, providing a standardized measure for the assessment of characteristics of tinnitus sound in clinical practice.

## 1. Introduction

Tinnitus is a highly heterogeneous condition both in terms of its origin as well as its manifestation [1]. There are various causes of tinnitus, including ear and hearing problems, exposure to noise or ototoxic medication, stress, and various health conditions such as neurological conditions. Tinnitus can also be highly diverse in terms of perception and associated consequences (e.g., pitch, loudness, how many different sounds are heard and/or noticed), which can vary substantially between and within individuals. Moreover, experiences of tinnitus may also vary, with most individuals living a good life with minor inconveniences, while some may experience, related by patient reports of the sound experience itself, severe negative effects such as difficulty concentrating on a task, sleeping, anxiety, and depression [2].

Current tinnitus measures predominantly capture severity and impact and fail to provide a standardized method to characterize tinnitus sound perception [3]. Clinicians generally depend on subjective descriptions of tinnitus characteristics, asking questions about constant versus intermittent tinnitus, sudden onset versus gradual onset, duration, types of pitch (i.e., low vs. high), or how it may sound (e.g., ringing, buzzing, hissing) in an informal way, indicating a need for a structured, validated assessment tool. Moreover, while behavioral tests such as tinnitus pitch and loudness matching were more commonly used in tinnitus clinics and research several years ago [4], they are now less frequently employed. Although they may provide some insight into what tinnitus might sound like [5], they do not fully capture all aspects of its perceptual qualities. A study by Lentz and He [6] examined the perceptual dimensions of tinnitus using a multidimensional scaling method. The study results suggested pitch, modulation depth with spectral elements, and envelope rate as key elements employed by patients who tried to describe tinnitus sounds. A few earlier studies also examined individual descriptions of tinnitus to classify typical sounds reported by patients. For example, using the descriptions of 1625 tinnitus patients, Meikle et al. [7] found that most people described their tinnitus as ringing, hissing, clear tone, high-tension wire, or buzzing. These patient descriptions highlighted that the tinnitus sound may go beyond pitch or loudness and may include additional attributes.

A key focus during tinnitus assessment, both during initial diagnostic and intervention, is to measure tinnitus severity (or distress) using standardized and validated patient-reported outcome measures. The Tinnitus Functional Index (TFI; [8]) is a commonly used measure, and clinicians may also employ assessments of comorbidities such as anxiety and depression. Although such assessments focus on measuring the impact of tinnitus, they do not provide information regarding the tinnitus sensation itself. While the tinnitus pitch or loudness does not have strong correlation with tinnitus severity [9], the relationship between tinnitus acoustic characteristics (i.e., pitch, loudness, number of sounds) and tinnitus impact (i.e., tinnitus distress, anxiety, depression) has not been fully explored [10]. For instance, interactions between tinnitus and the auditory scene [11], and the role of attention [12] are related to the perception of the tinnitus sounds. Additionally, aspects such as hearing sensitivity and being able to hear sounds other than tinnitus play a role, as evidenced by the correlation between hearing levels and tinnitus distress [13]. Finally, although the relation between psychophysical measures of tinnitus and tinnitus severity is not strong, it is likely true that a person’s tinnitus would be more bothersome for that person if the sensation increased noticeably in loudness or spectral complexity over time.

In addition to existing tinnitus severity or distress measures such as TFI and Tinnitus Handicap Inventory (THI), there have also been attempts in the recent years from researchers around the world to develop new tinnitus measures [14,15]. However, these measures also elucidate the familiar construct of tinnitus severity and impact upon which several intake forms already focus [16]. We are unaware of any existing self-reported measures focusing on specifying the acoustic characteristics and qualities of a patient’s tinnitus other than commonly used visual analog scales.

Evidence-based tinnitus management options, including psychological-based therapies such as cognitive behavioral therapy (CBT) and sound therapy-based approaches (e.g., hearing aids or masking devices), focus on helping patients adapt to tinnitus, provide relief, and manage their condition successfully. However, many patients with tinnitus continue to seek a possible cure that could silence their tinnitus [17]. Although there is no known cure for tinnitus, it would be interesting to examine whether tinnitus interventions could modify tinnitus sound in a beneficial way for patients. For instance, whether (1) individuals with tinnitus who hear multiple sounds hear fewer sounds after intervention, (2) perceive their tinnitus as less loud, and/or (3) the constancy of tinnitus sound reduces. However, due to the lack of standardized measures for quantifying the tinnitus sound, these aspects have not yet been thoroughly investigated.

The aim of the current study was therefore to develop and validate the Tinnitus Qualities and Impact Questionnaire (TQIQ), a new tool for evaluating perceived qualities of tinnitus sound. An underlying assumption was that the patient-reported, subjective acoustic characteristics of tinnitus (i.e., tinnitus sound or quality) could be related to tinnitus severity. The TQIQ is designed to measure tinnitus sound (or quality) to understand its characteristics for an individual across various dimensions such as loudness, pitch, frequency, and maskability.

## 2. Method

### 2.1. Study Design

**Figure 1:**
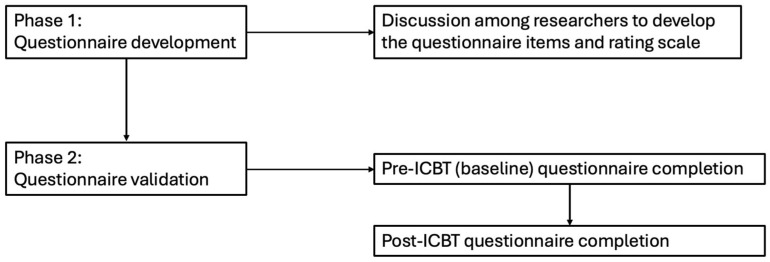
Flow diagram showing study phases and process.

The study was conducted in two phases. Phase I involved the development of the TQIQ, and Phase II involved assessing measurement properties of TQIQ (See Figure 1). The study was nested in several clinical trials (Clinical Trials.gov registration numbers: NCT04004260 and NCT04335812) on internet-based interventions for tinnitus [18,19,20]. The baseline data (cross-sectional) were collected before the internet-based cognitive behavioral therapy (ICBT) intervention and the post-data (longitudinal) included baseline and 8-weeks post intervention. Both cross-sectional as well as pretest–posttest data were used for the psychometric validation. The research was approved by the Institutional Review Board at Lamar University, Beaumont, TX, USA (IRB-FY17-209 on 7 June 2019 and IRB-FY20-200 on 2 April 2020). The COnsensus-based Standards for the selection of health Measurement INstruments (COSMIN) checklist [21,22] was used as a guide for reporting the study.

### 2.2. Participants

The study participants were drawn from larger clinical trials [18,19,20] and included those who were undergoing an eight-week internet-based cognitive behavioral therapy (ICBT) intervention. All the eligible participants from the clinical trials were included in the current study. The inclusion criteria for the study were adults aged ≥ 18 years who self-reported bothersome tinnitus. Participants needed internet access using either a smartphone or computer and were not receiving concurrent tinnitus therapy. A minimum sample size of 100 participants, or seven times the number of items in the questionnaire (i.e., 10 items × 7 = 70 participants), was indicated as sufficient for questionnaire validation studies [23]. In the current study, the aim was to include more than 100 individuals with tinnitus to ensure a sufficient sample size.

### 2.3. Data Collection

Online questionnaires were used throughout the study by all participants. Participants completed a baseline (or pre-intervention) demographic questionnaire that provided data on age, gender, employment status, ethnicity, and tinnitus duration. In addition, they completed a series of standardized, validated measures at baseline and immediately after completion of the ICBT intervention for tinnitus (i.e., post-intervention), including (a) tinnitus severity: TFI [8]; (b) anxiety symptoms: Generalized Anxiety Disorder-7 (GAD7; [24]); (c) depressive symptoms: Patient Health Questionnaire-9 (PHQ-9; [25]); (d) sleep disturbance: Insomnia Severity Index (ISI; [26]); and (e) health-related quality of life (HRQoL): EQ-5D-5L Visual Analog Scale (VAS) [27].

### 2.4. Data Analysis

To ensure that the TQIQ demonstrated sound psychometric properties, we followed published standards for assessing instruments underpinned by a formative model (COSMIN: [22,28]) and adhered to the criteria recommendations by Terwee et al. [23]. Psychometric testing was conducted on construct validity, internal consistency, floor and ceiling effects, interpretability, and responsiveness against established criteria, as outlined in the sections below. The data were analyzed using IBM SPSS Statistics for Windows Version 27.0. The analysis was conducted and reported in accordance with published recommendations [23].

Exploratory Factor Analysis (EFA). EFA was employed to provide information about the factor structure of the TQIQ. It is recommended that the minimum number of participants required for EPA is five participants per one item [29]. Therefore, a minimum sample size of 50 was considered adequate to perform EFA on the 10-item TQIQ. Several statistics were also inspected to ensure that the TQIQ data were suited to EFA. First, the Bartlett Test of Sphericity [30] was carried out, where a significant test (*p* < 0.05) is desirable. Second, the Kaiser Meyer Olkin (KMO; [31]) measure of sampling adequacy was examined to ensure that the sample size was appropriate. The KMO value should ideally be ≥0.80.

EFA was performed using Maximum Likelihood extraction and oblique rotation methods. The former is considered suitable when data are relatively normally distributed [32], with the latter also preferable as it was predicted that the TQIQ would comprise correlated factors. The procedure used to determine the number of factors to be extracted included a visual examination of the scree plot, which contains the number of factors on the *x*-axis and the corresponding eigenvalues on the *y*-axis. Eigenvalues are the percentage of variance accounted for by a factor. The number of factors to be extracted is the number of eigenvalues located before the “elbow-point” of the plot (i.e., the point at which there is a considerable decrease in the magnitude of the eigenvalues). The results of this approach were cross-checked with the results of another approach—extracting the smallest number of factors with the highest eigenvalues that cumulatively explain at least 50% of the variance [33]. The pattern matrix was examined as a means of exploring the potential factor structure of the TQIQ. The pattern matrix displays the factor loadings for each item, whereby the “cleanest” factor structure is the one where each item has a factor loading of ≥0.30 for a single factor [29]. In addition, the items should have no or few item cross-loadings, which occurs when items have factor loadings of ≥0.30 for more than one factor. To be considered stable, each factor should have a minimum of three items [29].

Construct Validity. According to Terwee et al. [23], construct validity can be assessed by testing specific, predefined hypotheses and is confirmed when at least 75% of *a priori* hypotheses are supported. First, we assessed convergent validity, which refers to the extent to which an instrument is correlated with other instruments that measure similar constructs. We conservatively predicted that TQIQ total scores would have a moderate, positive correlation (0.30 to 0.59) with the TFI, including all subscales. In addition, given that tinnitus severity has been shown to be associated with anxiety, depression, and general HRQoL [34], it was also predicted that the TQIQ would have at least moderate, positive correlations with GAD-7, PHQ-9, and ISI, and a moderate, negative correlation with EQ-5D-5L VAS. Pearson’s correlation coefficients (r) were used to test these predictions. Second, we assessed known-groups (or discriminative) validity, which refers to the ability of an instrument to distinguish between different subgroups [35]. We predicted that individuals with severe problems (TFI scores > 50; [8] would obtain significantly higher TQIQ total scores compared to those with a mild (TFI score < 25) or significant problem (TFI scores 25 to 50). An independent samples *t*-test was used to test this prediction.

Internal consistency reliability represents the extent to which items that purport to measure the same general construct produce similar scores. Cronbach’s alpha was used to assess this property, which should fall within the range of 0.70 to 0.95 for an instrument or its subscales [35]. Internal consistency was also assessed via the mean inter-item correlation, which should fall within the range of 0.30 to 0.70, and the mean corrected item total correlation, which should be ≥0.30 [36,37].

Floor and ceiling effects represents the proportion of respondents scoring the lowest (floor) or highest (ceiling) possible score on an instrument. Floor and ceiling effects are problematic as they suggest that an instrument is unable to differentiate between respondents at either extreme of the scale. Floor and ceiling effects were present if >15% of respondents achieved the lowest or highest possible score [23].

Interpretability indicates the degree to which qualitative meaning can be attributed to the quantitative scores of a measure [23]. This property was assessed using receiver operating characteristic (ROC) analyses, which assessed the ability of the TQIQ to accurately distinguish between categories of tinnitus severity as measured by the TFI [8]. Specifically, the TQIQ was assessed in terms of its ability to accurately differentiate between individuals with different tinnitus severity as (i) a mild problem (TFI score < 25); (ii) a significant problem (TFI score 25–50); or (iii) a severe problem (TFI score > 50).

ROC curves plotted sensitivity on the *y*-axis and specificity on the *x*-axis. The Area Under the ROC Curve (AUC) provided a global summary statistic representing the ability of the TQIQ to accurately discriminate between individuals in different tinnitus severity categories. An AUC of 0.5 means that there is a 50% probability that the measure cannot differentiate between two adjacent categories of patients. An AUC value ≥ 0.7 is considered desirable [36,37]. ROC analyses were also used to identify TQIQ cut-off scores for each category that had the optimal balance of sensitivity and specificity.

Responsiveness. This is also referred to as longitudinal validity and can be defined as the ability of an instrument to detect change over time [28]. According to Mokkink et al. [21], a construct approach to responsiveness can be tested by comparing change scores before and after intervention with other outcome measurement instruments. It was predicted that TQIQ change scores (i.e., the difference between pre- and post-ICBT intervention) would have a moderate, positive correlation with the TFI change scores, including all subscales. Pearson’s correlation coefficients (r) were used to assess this prediction.

Minimum Clinical Important Difference (MCID). This was estimated using the anchor-based method [38]. Specifically, given that it has been suggested that a reduction in TFI scores of 13-points should be meaningful to patients [8], mean TQIQ change scores (i.e., the difference between pre- and post-ICBT intervention) were stratified according to the following five groups: meaningfully worse (TFI change score ≥ −13), worse (−1 to −12), no change (0), better (1 to 12), and meaningfully better (≥13). The difference between TQIQ change scores for the “meaningfully better” and “unchanged” groups were then calculated and should be larger than at least one-half of the standard deviation (SD) for the initial TQIQ scores of the overall group [8].

## 3. Results

### 3.1. Phase I: Questionnaire Development

The questionnaire development was first conceptualized within the research team. An informal literature search was conducted to verify if any existing questionnaires were available that assess tinnitus sound qualities. As no suitable questionnaire instrument was found, another search was conducted to form perceptual dimensions of tinnitus [6,39]. To identify a comprehensive list of possible items, participants’ descriptions of tinnitus were collated from pre-trial questions such as “describe your tinnitus” [18,19,20]. These descriptions were analyzed recursively to identify a set of condensed categories capturing various dimensions of tinnitus sound qualities.

The identified categories were gradually refined by grouping similar descriptions and providing category labels. An initial key set of dimensions was selected by one author (EB). This list was then evaluated by a group of people with tinnitus who had previously undertaken the intervention and were part of a patient involvement advisory group for the intervention (*n* = 6). A draft questionnaire was produced with a condensed list of 10 items for the research team to review. This ensured the questionnaire was relevant and comprehensive and accurately measured the intended aspects of tinnitus. There were many considerations, particularly regarding the scoring.

Agreement was reached for the TQIQ to include 10-items, covering dimensions such as loudness, pitch, complexity, frequency, coexisting, distractability, maskability, mood, loud sounds, and sensitivity. A 10-item Likert scale (0 to 10) was adopted, which provides a score range of 0 to 100 (see Supplementary Table S1). A second optional section with four questions was developed to assess the respondents’ frequency of tinnitus awareness during different times of the day (i.e., morning, afternoon, evening, night), using a 5-point Likert scale (i.e., 0 = never aware to 4 = always aware).

### 3.2. Phase II: Psychometric Validation

In total, 308 participants with bothersome tinnitus completed all questionnaires and were included in the study. Table 1 shows that the mean age of participants was 55.5 years, a higher proportion identified as female (55%), were skilled/professional workers (58.1%), had at least a university degree (31.4%), were of white ethnicity (83.2%), and had experienced tinnitus for a mean duration of 12 years. While 240 (78%) individuals completed the ICBT intervention, 68 (22%) did not. Mean scores for frequency awareness of tinnitus were greatest in the evening (*mean* = 2.6; *SD* = 0.6), and lowest in the afternoon (*mean* = 2.3; *SD* = 0.7). See also Supplementary Table S2 for mean scores for each outcome measure.

### 3.3. Exploratory Factor Analysis

Bartlett’s Test of Sphericity was significant (*χ*^2^(45) = 1004.41, *p* < 0.001), leading to the rejection of the null hypothesis that the original correlation matrix is an identity matrix. In support of the adequacy of the sampling, the KMO value was 0.85. Together, both statistics demonstrated that it was appropriate to conduct EFA on the TQIQ data.

**Figure 2:**
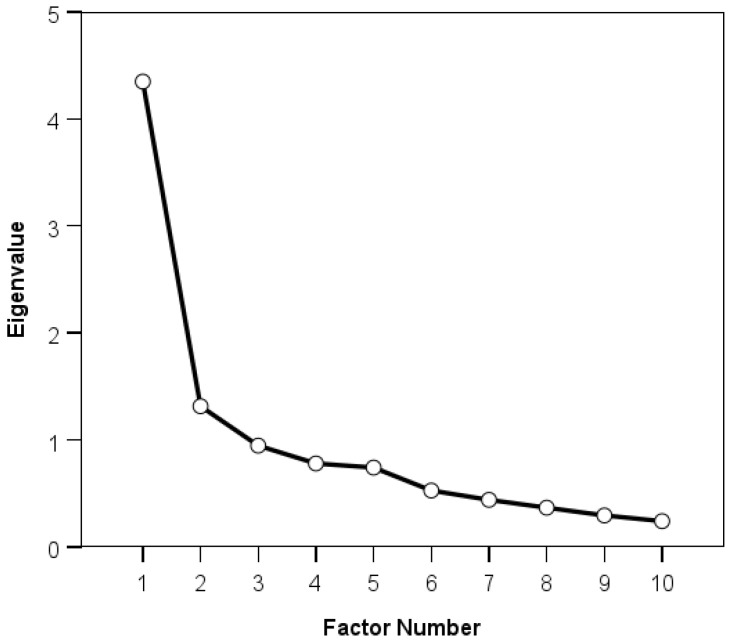
A scree plot depicting the relationship between the number of factors (*x*-axis) and the eigenvalues (*y*-axis).

Factor analysis identified two distinct factors—internal and external qualities of tinnitus—that together explained 56.64% of variance and suggested a clear structure. The inflection on the scree plot (Figure 2) occurred at five factors. Therefore, EFA was conducted separately for two-, three-, four-, and five-factor solutions. The two-factor solution offered the cleanest solution; all other factor solutions had fewer than three items with loadings of ≥0.3 and/or had multiple item cross-loadings (i.e., items had factor loadings of ≥0.3 for more than one factor). Factor one included six items (frequency, distractibility, maskability, pitch, loudness, coexisting), with factor loadings ranging from 0.69 to 0.80. This factor was termed “internal tinnitus qualities”, as the items center on the impact of internalized tinnitus qualities on the individual. Factor two included three items (loud sounds, sensitivity, mood), with factor loadings ranging from 0.36 to 0.79. This factor was termed “external tinnitus qualities”, as the items center on how external events/situations impact tinnitus qualities on the individual. One item (complexity) did not adequately load onto either factor, with factor loadings of 0.18 and 0.12 for factors one and two, respectively (Table 2).

### 3.4. Construct Validity

**Figure 3:**
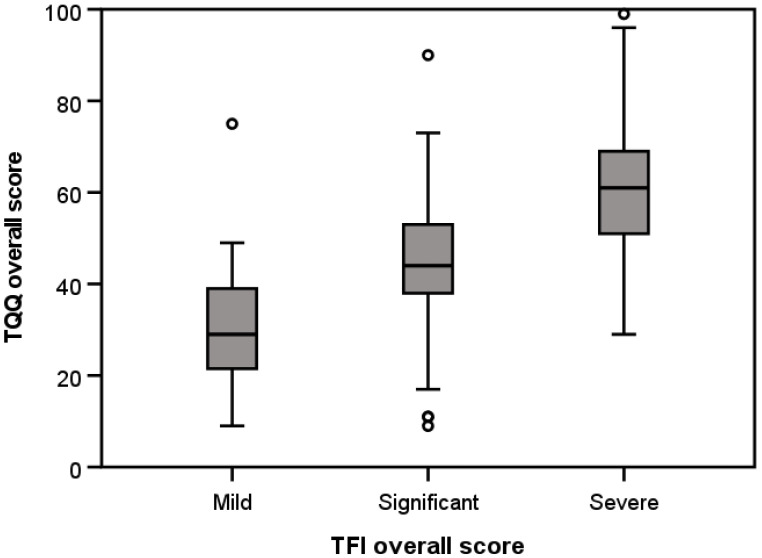
Boxplot showing overall scores for Tinnitus Qualities and Impact Questionnaire (TQIQ) for mild (<25), significant (25–50), and severe (>50) Tinnitus Functional Index (TFI) overall scores.

The TQIQ showed good construct validity, with 92% of predicted correlations confirmed. Total scores were significantly (i.e., strongly ≥0.60 or moderately 0.30 to 0.59) associated with tinnitus distress (TFI), anxiety (GAD-7), depression (PHQ-9), and insomnia (ISI) (Table 3). Similarly, the known-groups validity prediction was confirmed (Figure 3); individuals with an overall TFI score > 50 (severe) obtained significantly higher TQIQ scores (*mean* = 60.22; *SD* = 14.87; *n* = 138) compared to those with a score < 25 (mild) (*mean* = 31.48; *SD* = 3.10; *n* = 23), *t*(233) = −8.70, *p* < 0.001, or a score of 25–50 (significant) (*mean* = 44.35; *SD* = 13.83; *n* = 97), *t*(159) = −9.18, *p* < 0.001. Mild and significant TFI categories also differed significantly, *t*(118) = −3.96, *p* < 0.001.

### 3.5. Internal Consistency

All internal consistency statistics fell within the required range; the Cronbach’s α for the TOQ was 0.83, the mean corrected inter-item correlation was 0.35, and the mean corrected item-total correlation was 0.54 (Table 4).

### 3.6. Floor and Ceiling Effects

**Figure 4:**
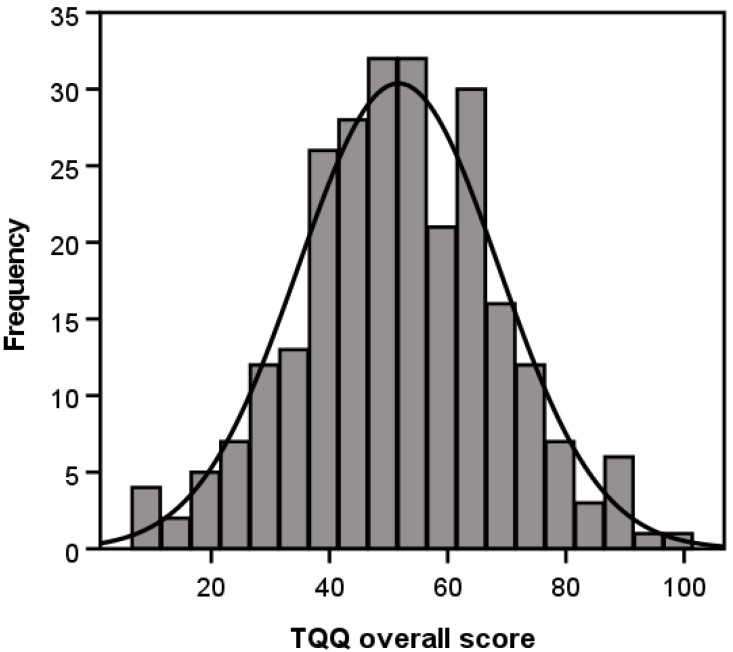
Distribution of Tinnitus Qualities and Impact Questionnaire (TQIQ) overall scores across all respondents.

TQIQ scores were normally distributed (Figure 4), with skewness of −0.001 (SE = 0.15) and kurtosis of 0.07 (*SE* = 0.30). No respondents scored the lowest (0) or highest (100) possible score (*mean* = 51.69; *SD*= 16.94; *range* = 9–99), suggesting that floor and ceiling effects were negligible.

### 3.7. Interpretability

**Figure 5:**
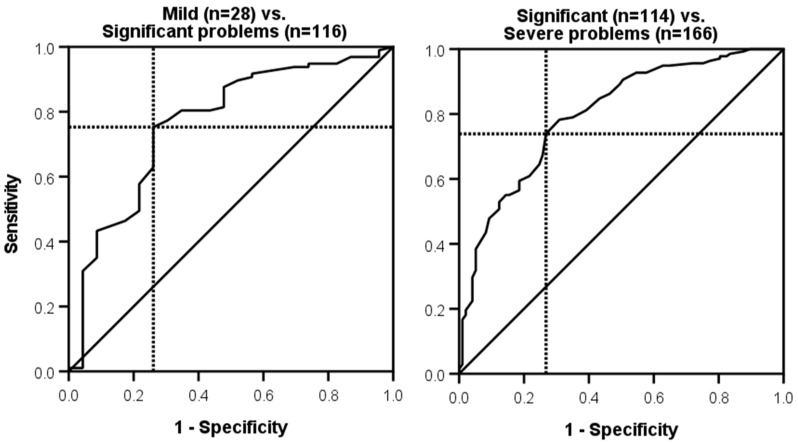
Receiver operating characteristic (ROC) curves for identifying the optimal cut-off scores for the TQIQ. Sensitivity is plotted on the *y*-axis and 1—specificity is plotted on the *x*-axis. The solid black line outlines the Area Under the ROC Curve (AUC). The solid line denotes 50% probability of accurately classifying tinnitus severity. The intersection of the broken black lines displays the cut-off score that provides an optimal balance of sensitivity and specificity.

The first ROC analysis (Figure 5) assessed the ability of the TQIQ overall score to accurately identify 28 individuals with tinnitus in the mild problems TFI category from 114 individuals in the significant problems category. The AUC was 0.765 (*95% CI* = 0.652–0.878, *p* < 0.001). A TQIQ cut-off score of 37.5 provided the best accuracy for the distinction between these two categories (sensitivity 75%, specificity 74%). The second ROC analysis assessed the ability of the TQIQ overall score to accurately identify 114 individuals with tinnitus in the significant problems TFI category from 166 individuals in the severe problems category. The AUC was 0.799 (*95% CI* = 0.742–0.856, *p* < 0.001). A TQIQ cut-off score of 51.1 provided the best accuracy for the distinction between these two categories (sensitivity 74%, specificity 73%).

### 3.8. Responsiveness

TQIQ was responsive, with change scores (i.e., the between pre- and post-ICBT intervention; *mean* = 10.74; *SD* = 15.74) showing moderate correlation with TFI changes, indicating sensitivity to treatment effects (Table 5).

### 3.9. Minimum Clinical Important Difference (MCID)

Table 6 presents the change in TQIQ scores in relation to TFI change scores (anchor). An ANOVA comparing TQIQ change scores by the TFI change group showed significant overall differences, *F*(4,135) = 5.44, *p* < 0.001. A reduction in TQIQ scores of around 19 points was estimated to be meaningful to patients; the mean change score for the “meaningfully better” group (15.2) compared with the “unchanged” group (−4.3) was about 19 points, which is larger than one-half of the SD observed for the initial TQIQ scores of the overall group (*Mean* = 51.69; *SD* = 16.94; *one-half SD* = 8.47). This difference between groups constitutes a large effect size, *d* = 1.44 [40].

## 4. Discussion

The current study outlined the development and psychometric validation of a new patient-reported measure (PROM) of tinnitus sound quality, namely, the TQIQ. EFA identified two factors accounting for 56.64% of the variance in the TQIQ data: “internal tinnitus qualities” (frequency, distractibility, maskability, pitch, loudness, coexisting) and “external tinnitus qualities” (loud sounds, sensitivity, mood). One item (complexity) did not adequately load onto either factor. Despite this, the TQIQ demonstrated adequate measurement properties (i.e., construct validity, internal consistency reliability, floor and ceiling effect, interpretability, and responsiveness) based on the pre-determined criteria that were developed using the guidelines by Terwee et al. [23] and the COSMIN [21,22].

The TQIQ is a concise, 10-item instrument designed for use in both clinical and research settings. It can be administered using either paper-and-pencil methods or digital platforms, making it versatile for in-person and virtual delivery. Given that it takes less than five minutes to complete, the TQIQ imposes minimal burden on respondents. We recommend administering the TQIQ alongside other established tinnitus questionnaires, such as the TFI or the Tinnitus Handicap Inventory (THI). While instruments like TFI and THI primarily assess tinnitus-related distress or severity, the TQIQ is specifically focused on evaluating the perceptual qualities of the tinnitus sound itself. Although these dimensions are related—as indicated by correlational analyses—they capture distinct aspects of the tinnitus experience and are therefore complementary in assessment.

### 4.1. Relations Between Tinnitus Severity and Tinnitus Qualities

Research shows a weak correlation between tinnitus severity and psychophysically measured acoustic characteristics, but more recent evidence suggests a nuanced relation [41]. For instance, a study by Meikle et al. [42], which included studies of 1800 patients in tinnitus clinic, found no correlation between tinnitus severity and tinnitus loudness, type, quality, or pitch when examined using behavioral tests. However, subsequent studies have suggested a more complex relation between tinnitus loudness and severity. For example, a large-scale (*n* = 4995) German study found that both tinnitus loudness and tinnitus annoyance were higher in individuals with binaural or centrally perceived tinnitus, increased noise sensitivity, and continuous tinnitus. A narrative synthesis of qualitative studies of patient reported complaints has also highlighted negative attributes of tinnitus sound (e.g., pitch, loudness, sound awareness, unpleasantness) as a key domain [43]. These findings suggest that physical attributes of tinnitus may influence tinnitus distress and annoyance. Considering the ecological framework outlined by Searchfield [11], it is likely that the complexity of the environment–tinnitus interaction is missed in the behavioral tests conducted in clinics. These findings suggest that the physical attributes of tinnitus may influence tinnitus distress and annoyance. Indeed, one could imagine asking a roomful of patients to raise their hand if they hear tinnitus. After all the hands go up, one might then ask, as Rich Tyler has in the past, “how many of you would not mind if your tinnitus suddenly got louder?” All the hands would likely go down; Tyler’s demonstration suggests that even among people not particularly bothered by tinnitus, an increase in loudness would be unwelcome.

It is generally accepted that the personal distress experienced by individuals with tinnitus is primarily mediated by acceptance, coping, and cognitive appraisal [44]. Therefore, we argue that the perceived attributes of tinnitus should not be neglected. These are the aspects that tinnitus patients often talk about when asked open-ended questions about tinnitus in a clinical context, encouraging them to describe problems that could be easily missed when using existing standardized PROMs [45]. TQIQ enables semi-structured clinical conversations around tinnitus sound perception and complements existing severity measurements. In the current study, tinnitus severity measured by the TFI, and the quality or physical attributes of tinnitus as perceived by individuals with tinnitus, showed moderate to strong correlations, supporting the hypothesis that tinnitus qualities and tinnitus severity are related. For this reason, using a standardized approach to assess tinnitus sound or quality using the self-reported TQIQ measure in addition to other existing behavioral measures may have some benefit in the clinical context [6].

### 4.2. Change in Tinnitus Qualities over Time or Following Intervention

Not everyone with tinnitus may experience distress, and even those who experience annoyance and distress in their early stages can adapt and experience less distress about their tinnitus over time [46]. Although poorly studied, some individuals with chronic tinnitus that has persisted for several years or decades may experience a complete disappearance of the sound [47,48]. Despite the natural course of adaptation, where many experiencing reduced tinnitus distress over time, the general consensus is that the acoustic characteristics of tinnitus (i.e., type of sound, loudness, laterality) largely remains stable over time for most individuals with chronic tinnitus [48].

TQIQ scores decreased after ICBT, and were associated with improvements on TFI, indicating potential sensitivity to therapeutic change. These results suggest that interventions could potentially alter tinnitus sensation and qualities. This further emphasizes the need for measuring and reporting tinnitus qualities in clinical trials that examine the efficacy and effectiveness of interventions, including psychological, pharmacological, and sound therapies, for tinnitus. Although many current and emerging interventions specify the need to psychophysically measure the items in the TQIQ, the TQIQ may provide analogous information without requiring the difficult and frustrating psychophysical measures. We have established MCID for the TQIQ using the study sample undergoing ICBT intervention. However, it is noteworthy that there is considerable variation in MCID established for several existing tinnitus outcome instruments [49]. This variation could be attributed to study population and the methodology employed. For this reason, further examination of MCID for TQIQ is needed to ensure if these values are replicable.

### 4.3. Strengths, Limitations, and Future Directions

The current study is likely one of the first to develop a standardized self-reported instrument to measure patients’ perceptions of tinnitus sounds and qualities. Individuals with tinnitus may find it valuable to have their tinnitus sound discussed and characterized in addition to counseling regarding the distress associated with having tinnitus. The study included an adequate sample size and pre-determined hypotheses for the psychometric validation. However, there are some key limitations. First, the questionnaire was developed based on an informal literature review and discussions among research team members. While the team primarily consisted of clinician–scientists who regularly provide care to individuals with tinnitus, this approach did not fully incorporate the patient perspective. As a result, the face validity of the questionnaire was only assessed informally within the research team and should be formally evaluated with external stakeholders in future studies. Second, the study sample included individuals seeking help and willing to participate in research assessing the effectiveness of an internet-based psychological intervention. Although the study sample included individuals with varied tinnitus severity and comorbidities, there were more individuals with higher severity of tinnitus than typically seen in a clinical sample, resulting in a potential sampling bias that limits generalizability. Repeating this study with a more representative clinical sample using random or consecutive sampling methods would be informative. Third, the study focused solely on the relationship between existing PROMs and did not include any behavioral measures of tinnitus sound such as tinnitus loudness and pitch matching. It would be interesting to examine the relationship between TQIQ and other behavioral measures such as tinnitus loudness matching and maskability. Fourth, while the questionnaire focused on qualities, in some items (e.g., pitch), the emphasis when interpreting results centers on annoyance or impact rather than the sound quality itself. Fifth, since participants completed all the questionnaires, their responses on the TQIQ may have been unduly influenced. Finally, it would be beneficial to explore how different tinnitus interventions may impact TQIQ in diverse tinnitus populations. Sixth, the current study did not include any cultural or linguistic adaptations to account for the diversity of populations, even within a single country such as the United States. Seventh, although several psychometric properties were assessed, test–retest reliability was not evaluated. Additionally, one item—”complexity”—did not load onto either of the two identified factors, which complicates the interpretation of the overall factor structure.

## 5. Conclusions

The current study reports that the newly developed TQIQ instrument focusing on tinnitus qualities (i.e., acoustic characteristics) has adequate psychometric properties in terms of factor structure, convergent validity, known-groups validity, internal consistency reliability, and floor and ceiling effect. TQIQ is the first standardized measure of tinnitus sound perception and is shown to have excellent correlation with recognized severity measures (i.e., TFI) and clinical utility. This instrument can be a good supplement in research and clinics to measure aspects of tinnitus that are often not well measured. The self-reported measures have the advantage of convenience and time when compared to laboratory psychoacoustic measures. However, the current study was performed on a homogeneous research population. Future research should validate TQIQ across diverse clinical populations and explore its utility in treatment monitoring.

## Figures and Tables

**Table 1:** Baseline demographic information of individuals with tinnitus who did and did not receive ICBT intervention.

Demographic Variable	Overall *n* = 308	Intervention *n* = 240	No Intervention *n* = 68
Age (years)			
Mean	55.5	54.7	58.4
SD	12.7	13.2	10.4
Range	19–84	19–81	34–84
Gender (*n*)			
Male	139 (45.1%)	108 (45.0%)	31 (45.6%)
Female	169 (54.9%)	132 (55.0%)	37 (54.4%)
Employment status (*n*)			
Entry level/unskilled work	10 (3.2%)	9 (3.8%)	1 (1.5%)
Skilled/professional work	179 (58.1%)	145 (60.4%)	34 (50.0%)
Retired	93 (30.2%)	68 (28.3%)	25 (36.8%)
Not working/unemployed	26 (8.4%)	18 (7.5%)	8 (11.8%)
Education (*n*)			
<High school	5 (1.6%)	5 (2.1%)	0 (0.0%)
High school	30 (9.7%)	20 (8.3%)	10 (14.7%)
Some college but not degree	84 (27.3%)	60 (25.0%)	24 (35.3%)
>University degree	189 (61.4%)	155 (64.6%)	34 (50.0%)
Ethnicity (*n*)			
American Indian/Alaska Native	3 (1.0%)	3 (1.3%)	0 (0.0%)
Asian	3 (1.0%)	2 (0.8%)	1 (1.5%)
Black/African American	7 (2.3%)	7 (2.9%)	0 (0.0%)
Native Hawaiian/Other Pacific Islander	1 (0.3%)	1 (0.4%)	0 (0.0%)
White Hispanic/Latino	47 (16.8%)	39 (18.2%)	8 (12.3%)
White Non-Hispanic/Latino	232 (83.2%)	175 (81.8%)	57 (87.7%)
More than One Race	15 (4.9%)	13 (5.4%)	2 (2.9%)
Tinnitus duration (years)			
Mean	12.3	12.0	13.4
SD	13.1	13.3	12.5
Range	<1–70	<1–70	<1–58
Frequency awareness of tinnitus, mean (SD)			
Morning	2.5 (0.7)	2.5 (0.8)	2.6 (0.7)
Afternoon	2.3 (0.7)	2.5 (0.7)	2.3 (0.7)
Evening	2.6 (0.6)	2.7 (0.5)	2.6 (0.6)
Night	2.3 (0.9)	2.5 (0.7)	2.3 (0.9)

**Table 2:** Pattern matrix factor loadings for each TQIQ item. Bold indicates factor loadings ≥ 0.3.

TQIQ Domain	TQIQ Question	Factor 1	Factor 2
Frequency	How often are you aware of your tinnitus?	**0.80**	−0.16
Distractibility	How much do you notice your tinnitus when you are busy doing other things?	**0.76**	0.05
Maskability	How much do you notice your tinnitus when there are other sounds around you?	**0.74**	0.04
Pitch	How annoyed are you with the pitch (or tone) of your tinnitus?	**0.74**	−0.02
Loudness	How loud has your tinnitus been?	**0.72**	−0.02
Coexisting	How easily have you lived with having tinnitus?	**0.69**	0.14
Complexity	How many different types of sound do you hear?	0.18	0.12
Loud Sounds	How has hearing loud noise affected your tinnitus?	−0.05	**0.79**
Sensitivity	How sensitive are you to sounds you hear around you?	0.01	**0.60**
Mood	How much does your mood affect your tinnitus?	0.28	**0.36**

TQIQ, Tinnitus Qualities and Impact Questionnaire.

**Table 3:** Pearson’s correlation coefficients to test convergent validity for Tinnitus Qualities and Impact Questionnaire (TQIQ).

Instrument	*r*	*p*
TFI Overall	0.70	<0.001
TFI Intrusive subscale	0.61	<0.001
TFI Sense of Control subscale	0.52	<0.001
TFI Cognitive subscale	0.61	<0.001
TFI Sleep subscale	0.48	<0.001
TFI Auditory subscale	0.38	<0.001
TFI Relaxation subscale	0.56	<0.001
TFI Quality of Life subscale	0.61	<0.001
TFI Emotional subscale	0.59	<0.001
GAD-7	0.49	<0.001
PHQ-9	0.49	<0.001
ISI	0.43	<0.001
EQ-5D-5L VAS	−0.24	<0.001

TFI, Tinnitus Functional Index; GAD-7, Generalized Anxiety Disorder-7 item; PHQ-9, Patient Health Questionnaire-9 item; ISI, Insomnia Severity Index; VAS, Visual Analog Scale.

**Table 4:** Corrected inter-item and item-total correlations for each Tinnitus Qualities and Impact Questionnaire (TQIQ) question.

TQIQ Item	Mean (SD)	Inter-Item Correlation										Corrected Item-Total Correlation
		1	2	3	4	5	6	7	8	9	10	
1. Loudness	7.14 (2.08)	1.0										0.63
2. Pitch	6.91 (2.57)	0.69	1.0									0.62
3. Complexity	1.75 (2.45)	0.23	0.15	1.0								0.24
4. Frequency	7.08 (2.25)	0.55	0.52	0.11	1.0							0.56
5. Co-existing	5.22 (2.54)	0.53	0.58	0.19	0.48	1.0						0.70
6. Distractibility	4.02 (2.66)	0.44	0.49	0.15	0.59	0.62	1.0					0.67
7. Maskability	4.43 (2.74)	0.49	0.48	0.25	0.54	0.55	0.73	1.0				0.66
8. Mood	3.88 (3.07)	0.29	0.33	0.15	0.25	0.48	0.34	0.31	1.0			0.48
9. Lound sounds	5.41 (3.32)	0.21	0.23	0.16	0.14	0.32	0.30	0.28	0.34	1.0		0.43
10. Sensitivity	5.85 (2.81)	0.26	0.22	0.09	0.16	0.25	0.21	0.23	0.31	0.48	1.0	0.39

**Table 5:** Pearson’s correlation coefficients to test responsiveness for TQIQ and TFI change scores. Bold indicates moderate correlations (0.30 to 0.59).

Instrument	Mean Change (SD)	*r*	*p*
TFI Overall	21.07 (20.54)	**0.55**	**<0.001**
TFI Intrusive subscale	18.78 (21.76)	**0.44**	**<0.001**
TFI Sense of Control subscale	24.68 (26.47)	**0.51**	**<0.001**
TFI Cognitive subscale	19.17 (26.46)	**0.48**	**<0.001**
TFI Sleep subscale	22.60 (27.85)	**0.34**	**<0.001**
TFI Auditory subscale	16.22 (27.55)	0.26	<0.001
TFI Relaxation subscale	25.90 (26.95)	**0.46**	**<0.001**
TFI Quality of Life subscale	20.61 (26.35)	**0.51**	**<0.001**
TFI Emotional subscale	20.77 (24.72)	**0.46**	**<0.001**

TFI, Tinnitus Functional Index.

**Table 6:** Mean Tinnitus Qualities and Impact Questionnaire (TQIQ) change scores in relation to Tinnitus Functional Index (TFI) change scores. TQIQ change scores are stratified according to following five groups: meaningfully worse (TFI change score ≥ −13), worse (−1 to −12), no change (0), better (1 to 12), and meaningfully better (≥13).

	TQIQ Difference	
	Mean	SD
Meaningfully worse (≥−13)	3.0	11.8
Worse (−1 to −12)	−1.8	9.8
No change	−4.3	10.5
Better (1 to 12)	8.2	12.1
Meaningfully better (≥13)	15.2	16.0

## Data Availability

The data that support the findings of this study are openly available in Figshare at: http://doi.org/10.6084/m9.figshare.13681924 (accessed on 4 November 2024).

## Supplementary Materials

The following supporting information can be downloaded at: https://www.mdpi.com/article/10.3390/clinpract15050087/s1. Table S1: The Tinnitus Qualities and Impact Questionnaire (TQIQ); Table S2: Mean (standard deviation) baseline scores for each outcome measure for those who did and did not receive the ICBT intervention.
