# Supplementary material for: Development and Psychometric Validation of Tinnitus Qualities and Impact Questionnaire
Source: Clin Pract. 2025 Apr 27;15(5):87. doi: 10.3390/clinpract15050087 (PMC12110700; doi:10.3390/clinpract15050087)
Supplement: Supplementary file 1 [file clinpract-15-00087-s001.zip › clinpract-3510602-supplementary.pdf]

Supplementary Table S1: The Tinnitus Qualities and Impact Questionnaire (TQIQ)

Instructions: Please respond to the following questions. Select only one of the numbers from 0 to 10 for each question. Select a lower score (0–3) when an aspect has not been a problem. Select a higher number (7–10) when an aspect has been a big problem. If it is only a moderate problem, select a middle number (4–6).

**Domain**	**Question**	**Select one number for each question.**										
Loudness	1. How loud has your tinnitus been?	0 = Hardly noticeable	1	2	3	4	5	6	7	8	9	10 = Very loud
Pitch	2. How annoyed are you with the pitch (or tone) of your tinnitus?	0 = Not at all	1	2	3	4	5	6	7	8	9	10 = Very annoyed
Complexity	3. How many different types of sound do you hear?	0 = A single sound	1	2	3	4	5	6	7	8	9	10 = More sounds than I can count (10+)
Frequency	4. How often are you aware of your tinnitus?	0 = Rarely aware	1	2	3	4	5	6	7	8	9	10 = Always aware
Coexisting	5. How easily have you lived with having tinnitus?	0 = Very easily	1	2	3	4	5	6	7	8	9	10 = Really struggling

Distractibility	6. How much do you notice your tinnitus when you are busy doing other things?	0 = Hardly noticeable	1	2	3	4	5	6	7	8	9	10 = Notice it a lot
Maskability	7. How much do you notice your tinnitus when there are other sounds around you?	0 = Hardly notice it	1	2	3	4	5	6	7	8	9	10 = Notice it a lot
Mood	8. How much does your mood affect your tinnitus?	0 = Not at all	1	2	3	4	5	6	7	8	9	10 = Very much
Loud sounds	9. How has hearing loud noise affected your tinnitus?	0 = Not at all	1	2	3	4	5	6	7	8	9	10 = Very much
Sensitivity	10. How sensitive are you to sounds you hear around you?	0 = Not at all	1	2	3	4	5	6	7	8	9	10 = Very sensitive
	**How to score?** Add all the scores for questions 1 through 10. Add the weekly score to Table 28-3. Lower scores indicate fewer problems. These scores should decrease over time.											

Frequency of Tinnitus Awareness Questionnaire

Please rate how aware you are of your tinnitus. Select only one of the numbers between 0 and 4 for each question. Select lower scores (0–2) when you are not very aware of your tinnitus. Select higher numbers (3–4) when you are frequently aware of your tinnitus.

	**0 = Never aware**	**1 = Seldom aware**	**2 = Sometimes aware**	**3 = Often aware**	**4 = Always aware**
1. In the morning					
2. In the afternoon					
3. In the evening					
4. During the night					
**How to score?**<br>Add all the scores for questions 1 through 4. Lower scores indicate fewer problems. These scores should decrease over time.					

Scoring: The scores are calculated by adding responses of the 10 items in the main scale. The scores can range from 0 to 100. Scores from 0-37 suggest a minimal effect on tinnitus qualities, 38-51 suggest a moderate effect, and scores ≥52 suggest a severe effect on tinnitus qualities. A pre-post reduction of 19 points is considered as a minimum clinically meaningful change.

Supplementary Table S2. Mean (standard deviation) baseline scores for each outcome measure for those who did and did not receive the ICBT intervention.

**Outcome measure**	**Overall** *N*= **308**	**Intervention** *n*= **240**	**No Intervention** *n*= **68**
TQIQ Overall	51.7 (16.9)	52.2 (16.9)	49.8 (17.2)
TFI Overall	53.1 (21.2)	52.7 (22.2)	54.4 (17.6)
TFI Intrusive subscale	65.0 (22.1)	63.6 (22.5)	70.2 (20.3)
TFI Sense of Control subscale	63.5 (21.)_	63.1 (22.0)	64.8 (18.2)
TFI Cognitive subscale	46.6 (26.2)	46.8 (27.0)	46.0 (23.6)
TFI Sleep subscale	49.3 (31.7)	49.2 (32.5)	49.6 (29.3)
TFI Auditory subscale	51.6 (29.4)	48.9 (29.4)	61.1 (27.5)
TFI Relaxation subscale	65.3 (27.9)	64.6 (29.0)	67.9 (23.7)
TFI Quality of Life subscale	42.8 (28.8)	43.3 (29.2)	40.9 (27.6)
TFI Emotional subscale	44.1 (28.9)	45.5 (29.7)	39.2 (25.5)
GAD-7	7.0 (5.4)	7.5 (5.6)	5.4 (4.2)
PHQ-9	7.1 (5.7)	7.5 (6.0)	5.6 (4.1)
ISI	11.3 (6.6)	11.6 (6.7)	10.0 (5.8)
EQ-5D-5L VAS	74.6 (15.5)	74.2 (15.9)	76.0 (12.2)

TQIQ, Tinnitus Qualities and Impact Questionnaire; TFI, Tinnitus Functional Index; GAD-7, Generalised Anxiety Disorder-7 item; PHQ-9, Patient Health Questionnaire-9 item; ISI, Insomnia Severity Index; VAS, visual analogue scale.
